# Revolutionising Cancer Immunotherapy: Advancements and Prospects in Non‐Viral CAR‐NK Cell Engineering

**DOI:** 10.1111/cpr.13791

**Published:** 2024-12-27

**Authors:** Zhaokai Zhou, Yifeng Chen, Yuhao Ba, Hui Xu, Anning Zuo, Shutong Liu, Yuyuan Zhang, Siyuan Weng, Yuqing Ren, Peng Luo, Quan Cheng, Lulu Zuo, Shanshan Zhu, Xing Zhou, Chuhan Zhang, Yukang Chen, Xinwei Han, Teng Pan, Zaoqu Liu

**Affiliations:** ^1^ Department of Interventional Radiology The First Affiliated Hospital of Zhengzhou University Zhengzhou China; ^2^ Department of Urology The First Affiliated Hospital of Zhengzhou University Zhengzhou China; ^3^ The First Affiliated Hospital of Zhengzhou University Zhengzhou China; ^4^ Department of Respiratory and Critical Care Medicine The First Affiliated Hospital of Zhengzhou University Zhengzhou China; ^5^ The Department of Oncology Zhujiang Hospital, Southern Medical University Guangzhou China; ^6^ Department of Neurosurgery Xiangya Hospital, Central South University Changsha China; ^7^ Center of Reproductive Medicine The First Affiliated Hospital of Zhengzhou University Zhengzhou China; ^8^ Department of Gastroenterology The First Affiliated Hospital of Zhengzhou University Zhengzhou China; ^9^ Department of Pediatric Surgery The First Affiliated Hospital of Zhengzhou University Zhengzhou China; ^10^ Department of Oncology The First Affiliated Hospital of Zhengzhou University Zhengzhou China; ^11^ Interventional Institute of Zhengzhou University Zhengzhou China; ^12^ Interventional Treatment and Clinical Research Center of Henan Province Zhengzhou China; ^13^ Longgang District Maternity & Child Healthcare Hospital of Shenzhen City (Longgang Maternity and Child Institute of Shantou University Medical College) Shenzhen China; ^14^ Institute of Basic Medical Sciences Chinese Academy of Medical Sciences and Peking Union Medical College Beijing China

**Keywords:** chimeric antigen receptor, immunotherapy, natural killer cell, non‐viral transfection, tumour microenvironment

## Abstract

The recent advancements in cancer immunotherapy have spotlighted the potential of natural killer (NK) cells, particularly chimeric antigen receptor (CAR)–transduced NK cells. These cells, pivotal in innate immunity, offer a rapid and potent response against cancer cells and pathogens without the need for prior sensitization or recognition of peptide antigens. Although NK cell genetic modification is evolving, the viral transduction method continues to be inefficient and fraught with risks, often resulting in cytotoxic outcomes and the possibility of insertional mutagenesis. Consequently, there has been a surge in the development of non‐viral transfection technologies to overcome these challenges in NK cell engineering. Non‐viral approaches for CAR‐NK cell generation are becoming increasingly essential. Cutting‐edge techniques such as trogocytosis, electroporation, lipid nanoparticle (LNP) delivery, clustered regularly interspaced short palindromic repeats–associated protein 9 (CRISPR‐Cas9) gene editing and transposons not only enhance the efficiency and safety of CAR‐NK cell engineering but also open new avenues for novel therapeutic possibilities. Additionally, the infusion of technologies already successful in CAR T‐cell therapy into the CAR‐NK paradigm holds immense potential for further advancements. In this review, we present an overview of the potential of NK cells in cancer immunotherapies, as well as non‐viral transfection technologies for engineering NK cells.

## Graphical abstract

This manuscript provides a comprehensive overview of the state‐of‐the‐art in non‐viral CAR‐NK cell engineering, highlighting its potential to revolutionise cancer treatment.

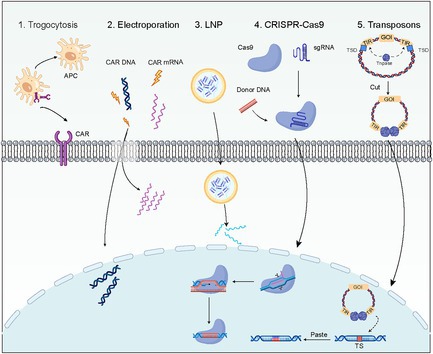

**Abbreviations**
ACT: adoptive cell therapy
AI: artificial intelligence
AML: acute myeloid leukaemia
APCs: antigen‐presenting cells
BBB: blood–brain barrier
BCMA: B‐cell maturation antigen
CAR: chimeric antigen receptor
CCR7: C–C chemokine receptor type 7
CRISPR‐Cas9: Clustered regularly interspaced short palindromic repeats–associated protein 9
CRS: cytokine release syndrome
DMSO: dimethyl sulfoxide
EP: electroporation
FDA: U.S. Food and Drug Administration
GvHD: graft‐versus‐host disease
HLA: human leukocyte antigen
IL: interleukin
iPSC: induced pluripotent stem cell
iPSC‐NK: induced pluripotent stem cell–differentiated CAR‐NK
KIR^+^
: killer immunoglobulin‐like receptor positive
LNP: lipid nanoparticle
MHC: major histocompatibility complex
MM: multiple myeloma
NK: natural killer
NKG2A: natural killer group 2A
NKG2D: natural killer group 2D
NNs: nanoneedles
PB: piggyBac
PD‐1: programmed cell death protein‐CIK1
R/R: relapsed or refractory
SB: sleeping beauty
siRNA: small interfering RNA
sNKG2D‐L: soluble natural killer group 2D ligands
TAA: tumour‐associated antigens
TB: TcBuster
TME: tumour microenvironment
VNB: vapour nanobubble

## 1 Introduction

Genetic modification stands as an ideal approach to implement anticancer therapy as it enables the production of personalised antitumour immune cells. Chimeric antigen receptor (CAR)–engineered natural killer (NK) cells could express CARs on their surfaces to recognise and target tumour cells with specific antigens [1]. CAR‐NK cells have several advantages over other types of CAR cells, including the potential to generate an ‘off‐the‐shelf’ product, none developed cytokine release syndrome (CRS) or neurotoxicity, and their ability to recognise and kill cancer cells without prior exposure to tumour‐associated antigens (TAAs). CAR‐NK cells have made significant advances in both treating haematological and solid tumours, holding promise as potent cancer immunotherapy [2]. In addition, the conduct of numerous preclinical and clinical trials has also underlined the substantial promise of CAR‐NK cell therapy [3, 4]. Overall, the advancement of CAR‐NK cell technology marks a significant milestone in cancer immunotherapy, harbouring the potential to revolutionise cancer treatment.

**FIGURE 1:**
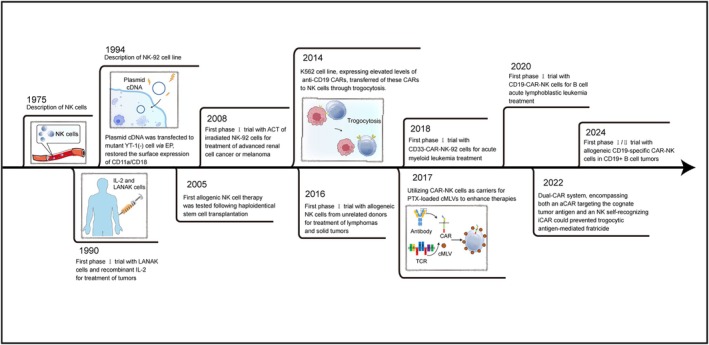
The history of NK cell–based immunotherapies. Key discoveries, important clinical trials, and highlights that were prominent in the last decades are illustrated. NK: natural killer; LANAK: lymphokine‐activated natural killer; IL‐2: interleukin‐2; ACT: adoptive cell therapy; CAR: chimeric antigen receptor; CD: cluster of differentiation; PTX: paclitaxel; cMLVs: cross‐linked multilamellar liposomal vesicles.

Although numerous efforts have been made to explore more efficacious treatments, the incidence and mortality rates of cancer patients continue to rise. Immuno‐ and gene therapy might be a way out of this dilemma. Genome editing tools could be effectively transported into cells via non‐viral gene delivery systems with a safety profile. Non‐viral vectors play an important role in cancer immunotherapy, including trogocytosis, electroporation (EP) and lipid nanoparticle (LNP). These vectors could be used to introduce CAR genes into NK cells, thus enhancing the antitumour effect. The gene editing technology can be harnessed to fight against tumours in cancer immunotherapy. Clustered regularly interspaced short palindromic repeats–associated protein 9 (CRISPR‐Cas9) technology, editing genes in immune cells, may be beneficial in improving the efficacy of cancer immunotherapy [5]. Furthermore, in contrast to the majority of methods that result in transient expression of CAR, transposon systems offer the simplest non‐viral gene delivery technology for achieving long‐term stable expression of CAR. We summarise the history of NK cell–based immunotherapies and illustrate this in Figure 1.

CAR‐NK engineering is a novel cancer immunotherapy strategy with a natural killing ability and lower toxicity [6]. Unlike CAR‐T cells, CAR‐NK cells circumvent the need for human leukocyte antigen (HLA) matching and can therefore be used as an ‘off‐the‐shelf’ treatment option with wider applicability. Non‐viral vectors could introduce CAR genes into NK cells to boost their antitumour immunity. Moreover, non‐viral vectors can ensure the safety of CAR‐NK cells. Herein, we summarised the strengths and weaknesses of cells with CAR–based immunotherapy and the current development of non‐viral vectors and gene editing technology for CAR‐NK cells, including trogocytosis, EP, LNP, CRISPR‐Cas9 and transposons. Ultimately, we discuss other non‐viral intracellular delivery techniques as promising points for further research into CAR‐NK cell therapy.

## 2 Development of CAR Engineering Technology

### 2.1 CAR‐T Cell

The adoptive transfer of T cells expressing CAR is a promising approach for cancer immunotherapy [7, 8]. This technique has demonstrated the potential to enhance patient outcomes. CAR‐T cells do not rely on the expression of major histocompatibility complex (MHC) or MHC homogeneity. Additionally, the injection of CAR gene–modified T cells has led to substantial clinical improvements, resulting in cancer remission for patients battling relapsed or refractory (R/R) malignancies [9]. Currently, the production of CAR‐T cells predominantly utilises γ‐retroviral or lentiviral vectors. However, these methods come with inherent limitations [10]. The U.S. Food and Drug Administration (FDA) declared it had received reports of T‐cell malignancies, including T‐cell large granular lymphocytosis, T‐cell lymphoma, cutaneous T‐cell lymphoma and peripheral T‐cell lymphoma, following treatment with all currently approved B‐cell maturation antigen (BCMA)– and CD19–directed genetically modified autologous CAR T‐cell immunotherapies [11, 12]. Among them, there have been leukaemia case reports confirming the presence of CAR genes in cancerous T cells, suggesting that CAR‐T products are likely to be involved in the development of T‐cell malignancies [12, 13]. The efficacy of CAR‐T therapy faces challenges owing to tumour resistance driven by antigen escape and potentially lethal side effects like CRS [14, 15]. The precise reasons behind the suboptimal performance of CAR‐T cells in solid tumours are not fully understood, largely due to the reduced antitumor activity of CAR‐T cells and the uncertainty of trafficking them to solid tumours. In solid tumours, it remains unclear whether the number of CAR‐T cells in the blood correlates with the number in solid tumours owing to limited migratory ability into tumours [16]. Notably, most clinical investigations of CAR T‐cell therapy in solid tumours have primarily focused on documenting general tumour response parameters and the detection or persistence of CAR‐T cells in peripheral blood [17, 18]. Critical insights into the infiltration, phenotype and interactions of CAR‐T cells with the tumour microenvironment (TME) remain largely unexplored.

#### 2.1.1 Non‐Viral Transfection of CAR‐T Immunotherapy

Retroviral vectors, both lentiviral and γ‐retroviral vectors, enhancing the in vitro genetic modification of T cells, have been used in FDA–approved CAR T‐cell products and the majority of current clinical trials [10, 19]. However, CAR T‐cell engineering is associated with various challenges, such as safety issues, high manufacturing costs and vector capacity constraints. Of note, despite CAR‐T products being associated with fewer cancers compared to the products derived from earlier generations of viruses utilised for gene therapy transduction, there remains a latent risk of oncogenesis attributable to genomic integration or alternative mechanisms inherent in the contemporary generation of retroviral vectors [20]. For example, lentiviral vector constructs, while integrating into the genome in a semi‐random manner, display a propensity to target regions of active gene expression within the genome, potentially raising concerns for insertional oncogenesis [21]. The intrinsic challenges associated with viral vectors spurred the exploration of non‐viral transfection techniques for ex vivo T‐cell modification. Currently, T‐cell transfection techniques encompass EP, sonoporation and the use of lipid and polymer nanocarriers. Some researchers introduced a novel approach for crafting gene‐specific targeted CAR‐T cells, an anti‐CD19 CAR cell with programmed cell death protein‐1 (PD‐1) integration via the CRISPR‐Cas9 system. In clinical trials, patients with R/R–aggressive B‐cell non‐Hodgkin lymphoma experienced enhanced safety when treated with non‐viral, PD‐1–integrated anti‐CD19 CAR‐T cells. These researchers showed that these CAR‐T cells remained effective even at low infusion doses and with a low percentage of CAR^+^ cells [22]. Although modified T cells with viral vectors promote the success of CAR T‐cell therapies, issues such as tumour relapse, intricate manufacturing processes and the expansion to treat other conditions like solid tumours demand more sophisticated design protocols and gene transfer technologies. Non‐viral technologies are rapidly evolving to address these challenges.

### 2.2 CAR‐NK Cell

NK cells' efficiency and their inherent advantages have led to their consideration as a candidate for genetic modification to generate effective effector cells for adoptive cell therapy (ACT) in cancer patients [23]. The basis of CAR T‐cell research provides insights into CAR‐NK development. CAR‐NK cell therapy has been acknowledged as a promising avenue in comparison with CAR T‐cell therapy (Table 1).

**TABLE 1:** Comparisons between CAR‐T cells and CAR‐NK cells.

	CAR‐T cells	CAR‐NK cells
Sources	Autologous or MHC–matched allogeneic	Autologous, non–MHC‐matched allogeneic or NK cell lines
Expansion	In vitro	Autologous NK cells expand in vitro. Cell like can also be pre‐expanded before transducing
Killing mechanisms	CAR–dependent cell killing	Both CAR–dependent and CAR–independent NK–mediated cell killing
Safety	GVHD; CRS; neurotoxicity; might cause T‐cell malignancies	Rarely cause GVHD; no CRS and neurotoxicity
Convenience	Less convenience	More convenience; The possibilities to be an ‘off‐the‐shelf’ product
Cost	$370,000–$530,000 per treatment in the USA	Potentially lower due to easier manufacturing
Current status	Six CD19 CAR T‐cell therapies have been approved by the FDA; new types of CAR‐T cells have been conducted in clinical trial	Preclinical and clinical trials have been conducted; several published data are available

Abbreviations: CAR, chimeric antigen receptor; CRS, cytokine release syndrome; GvHD, graft‐versus‐host disease; MHC, major histocompatibility complex; NK, natural killer.

#### 2.2.1 Advantages Over CAR‐T Cells

The primary advantage of CAR‐NK cells over CAR‐T cells lies in the cell source. The requirement for autologous T cells in CAR T‐cell therapy is due to the potential for alloreactivity and graft‐versus‐host disease (GvHD). Patients undergoing CAR‐T treatment often have low T cell counts as a result of prior heavy treatment, which may be a risk factor for inadequate collections [24]. CAR‐NK cells, however, do not require autologous NK cells for manufacturing. Therefore, CAR‐NK therapy avoids the challenges associated with the use of autologous cells.

Less side effects are also a major advantage. CAR T‐cell activation may lead to the release of vast amounts of inflammatory cytokines, triggering CRS and neurotoxicity. Results from a Phase I clinical trial demonstrated severe CRS in 14 of 53 patients after receiving CAR‐T cells manufactured at Memorial Sloan Kettering Cancer Center [25]. Similar toxicities were also observed in other clinical trials [26, 27]. In contrast, in a clinical trial, 11 patients with R/R cancers did not experience CRS or neurotoxicity when treated with HLA–mismatched anti‐CD19 CAR‐NK cells derived from cord blood [3]. The disparity in toxicity levels between CAR‐T and CAR‐NK cells might be attributed to variances in the cytokines released during cell activation. CAR T‐cell activation results in the release of multifarious cytokines and inflammatory markers, including tumour necrosis factor‐α, interleukin‐1β (IL‐1β), IL‐2 and IL‐6, whereas patients receiving CAR‐NK cell therapy did not show an increase in these inflammatory cytokines [3, 28]. The cytokines released by activated NK cells differ from the inflammatory cytokines released by T cells and do not cause high‐grade neurotoxicity [29].

In addition to the CAR–dependent pathway, NK cells possess inherent cytotoxic capabilities. Unlike T lymphocytes, which primarily rely on CAR‐specific targeting, NK cells possess natural cytotoxicity receptors permitting TAA–unrestricted target cell elimination. Additionally, the expression of IgG‐Fc–Receptor III family on NK cells facilitates antibody‐dependent cell‐mediated cytotoxicity. Hence, CAR‐NK cells offer both CAR–dependent and CAR–independent killing capacities, potentially outclassing CAR‐T in efficacy.

#### 2.2.2 Challenges Facing CAR‐NK Cell Therapy

The major challenge that CAR‐NK cell therapy faces is the limited in vivo lifespan of infused NK cells, thereby causing a deficiency in cytokine sustenance. The average lifespan of NK cells in the blood is generally around 2 weeks [30]. While this characteristic might be advantageous from a safety perspective with the ability to self‐regulate with the depletion of CAR‐NK cells provided on‐target off‐tumour toxicity occurred, it could hinder the effectiveness of CAR‐NK cell therapy. Exogenous cytokine administration has been shown to boost the proliferation and endurance of infused CAR‐NK cells. Nevertheless, this approach may also lead to undesirable consequences, including toxicities and a decrease in regulatory T cells [31, 32].

Encountering the suppressive TME might probably be the most significant obstacle to overcome in both CAR‐T and CAR‐NK therapies [33]. When CAR‐T cells enter the TME, the immunosuppressive environment hampers CAR‐T cells' function, which is particularly evident in certain cancers (*e.g.*, pancreatic cancer), where cellular and matrix components create a hostile microenvironment that hinders the efficacy of cancer immunotherapy [34]. In the case of NK cells, soluble factors further exacerbate their inhibition. Tumour cells could release soluble ligands, resulting in dysfunction of NK cells. Studies indicate that soluble NK group 2D ligands (sNKG2D‐L), a product of proteolytic shedding, downregulate NKG2D expression, consequently attenuating NK cell antitumour efficacy [35]. Similarly, BCL2–associated Athanogene 6‐soluble variant diminishes NK cell antitumour actions [36]. Of note, during ex vivo expansion, NK cells tend to express immune checkpoint receptors, potentially compromising CAR‐NK therapeutic potency [37]. Thus, understanding and overcoming this hostile TME remains paramount for advancing CAR‐NK therapy.

## 3 Development of Non‐Viral Vectors Regarding CAR‐NK Cell

### 3.1 Transient Non‐Viral Approaches in the Engineering Technology of CAR‐NK Cells

Currently, multiple techniques are available for transiently modifying NK cells via non‐viral methods, including trogocytosis, EP and LNP (Table 2). The common characteristic of these methodologies is their ability to facilitate the temporary expression of externally sourced proteins, with the objective of enhancing the overexpression of an additional receptor or protein.

**TABLE 2:** Current transient non‐viral technologies of CAR‐NK cells studies.

Transfection method	Published year	Source	Transfected CAR type	Efficiency	Reference
Trogocytosis	2009/11	Human KIR^+^ NK cell	CCR7	12%–37% (mean, 27.3%) (co‐cultured with dendritic cells)	[38]
Trogocytosis	2012/5	NK cells expanded from buffy coat–derived PBMCs	CCR7	80%	[39]
Trogocytosis	2014/10	NK (PBMCs)	Anti‐CD19	18.6% (1 h of co‐culture)	[40]
Electroporation	1994/6	YT‐1 NK cell line	CD18/cD11a	13%–99%	[41]
Electroporation	2009/9	NK‐92	Anti‐CD19	47.2% ± 8% (mRNA)/< 5% (cDNA)	[42]
Electroporation	2012/8	Primary NK	Anti‐CD19	28.2%–92.4% (median 61.3%) (24 h) (expanded NK cells)	[43]
Electroporation	2013	Primary NK	Anti‐CD19	NA	[44]
Electroporation	2019/4	NK‐92, resting or cytokine‐expanded human NK‐cell populations	Anti‐CD19, CCR7	∼40%/∼20%	[45]
Electroporation	2019/6	NK (PBMCs)	NKG2D	99%	[46]
Electroporation	2022/5	NK (PBMCs)	Anti‐BCMA/CXCR4	82%	[47]
Electroporation	2022/3	NK‐92 cell line	BCMA/CD19	88.1%	[48]
LNP	2017/12	NK‐92	Paclitaxel	NA	[49]
LNP	2023/4	NK‐92	NA	100%	[50]
LNP	2023/8	NK cells expanded from primary PBMCs	CD19, BCMA	78%, 95%	[51]
LNP	2023/9	NK (KHYG‐1), T (Jurkat)‐cell lines, cord blood–derived NK cells	NA	~100%	[52]

Abbreviations: BCMA, B‐cell maturation antigen; CAR, chimeric antigen receptor; CCR7, C–C chemokine receptor type 7; KIR^+^, killer immunoglobulin‐like receptor positive; LNP, lipid nanoparticle; NK, natural killer; PBMCs, peripheral blood mononuclear cells.

#### 3.1.1 Trogocytosis

**FIGURE 2:**
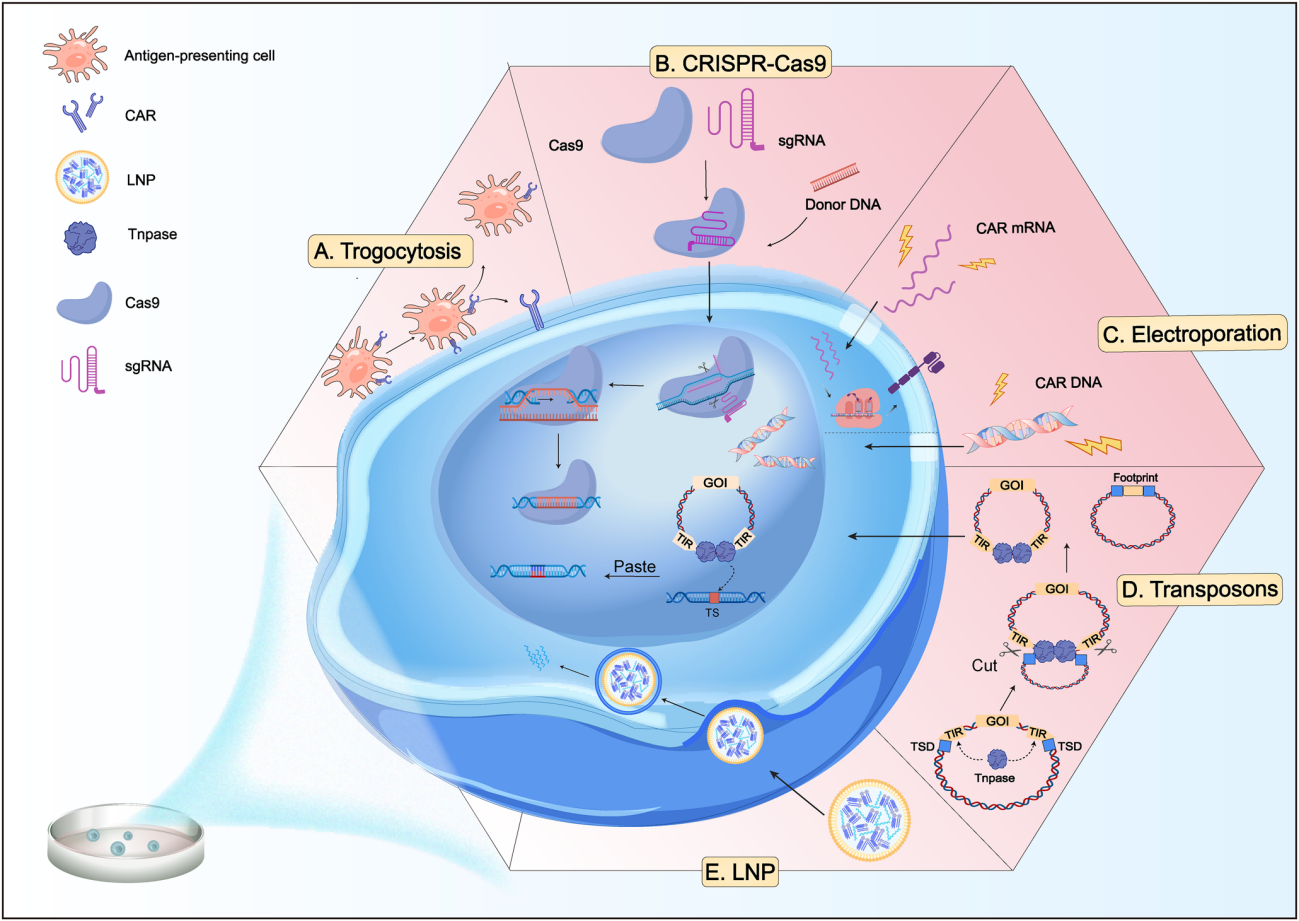
Illustration of different types of non‐viral technologies for NK cell engineering. (A). Trogocytosis: A process by which NK cells acquire membrane patches from APCs. The CAR is transferred from the APC to the NK cell, which enables functional modification. (B). CRISPR‐Cas9: A genome editing technique where the Cas9 protein, guided by sgRNA, introduces double‐stranded breaks in DNA. Donor DNA is subsequently integrated during the repair process, allowing for precise genomic insertion. (C). Electroporation: The transient formation of pores in cell membranes *via* electrical pulses facilitates the entry of CAR DNA and CAR mRNA directly into the cytoplasm. (D). Transposons: A transposase‐mediated ‘cut‐and‐paste’ mechanism, mobilised by transposase (Tnpase) molecules, can integrate a GOI into the host genome. The transposase binds to the TIRs, induces double‐stranded breaks, and excises the mobile element from the donor DNA leaving behind a footprint. The transposon–transposase complex finds a suitable TS and performs integration, producing a TSD. (E). LNP: LNP encapsulates RNA and facilitates its delivery into the cell, leveraging the cell's endocytic pathways for subsequent expression. APC: antigen‐presenting cell; sgRNA: single‐guide RNA; GOI: gene of interest; TIRs: terminal inverted repeats; TS: target site; TSD: target site duplication; LNP: lipid nanoparticle.

Introducing CAR constructs into NK cells is not solely restricted to traditional nucleic acids, it is also conveyed as a protein cargo via a mechanism termed trogocytosis as well [39, 40]. In the 1970s, the term ‘trogocytosis’ was used to characterise the process of the amoeba, *Naegleria fowleri* , nibbling and destroying other cells [53]. In 2002, the term was adapted to describe the transfer of associated surface molecules and plasma membrane fragments between cells (Figure 2A) [54]. Marcenaro and colleagues demonstrated in 2009 that both ‘licensed’ peripheral blood killer immunoglobulin‐like receptor–positive (KIR^+^) NK‐cell populations and KIR^+^ NK‐cell clones express C–C chemokine receptor type 7 (CCR7) de novo when co‐cultured with mature dendritic cells or Epstein–Barr virus‐transformed lymphoblastoid cell lines. Following co‐incubation with CCR7^+^ antigen‐presenting cells (APCs), the expression of CCR7 was found in NK cells, with protein level detection achievable for as long as 24 h [38]. Similar observations have been noted where NK cells quickly absorbed CCR7 from the engineered K562 cell line, though the effect persisted merely for only 3 days [39]. Cho et al. showed that a K562 cell line, which expressed elevated levels of anti‐CD19 CARs, transferred these CARs to NK cells through trogocytosis. Additionally, they revealed that co‐incubation with live APCs resulted in a more pronounced CAR expression, achieving a rate of 80%, compared to the 50% expression rate observed with nonviable K562 donor cells [40]. Furthermore, some researchers found that activated NK cells could acquire TYRO3 from tumour cells through trogocytosis, both in vitro and in vivo, leading to a marked increase in cytotoxicity and IFN‐γ production. When the APC K562 leukaemia cell line, a feeder cell line for NK cells expansion, overexpressed TYRO3, this receptor was transferred to NK cells through trogocytosis, subsequently enhancing NK cell expansion ex vivo. In comparison with TYRO3^−^NK cells, TYRO3^+^NK cells significantly enhanced IFN‐γ production, cytotoxicity and higher expression of activated surface markers [55]. This finding could provide invaluable insights into CAR engineering.

**FIGURE 3:**
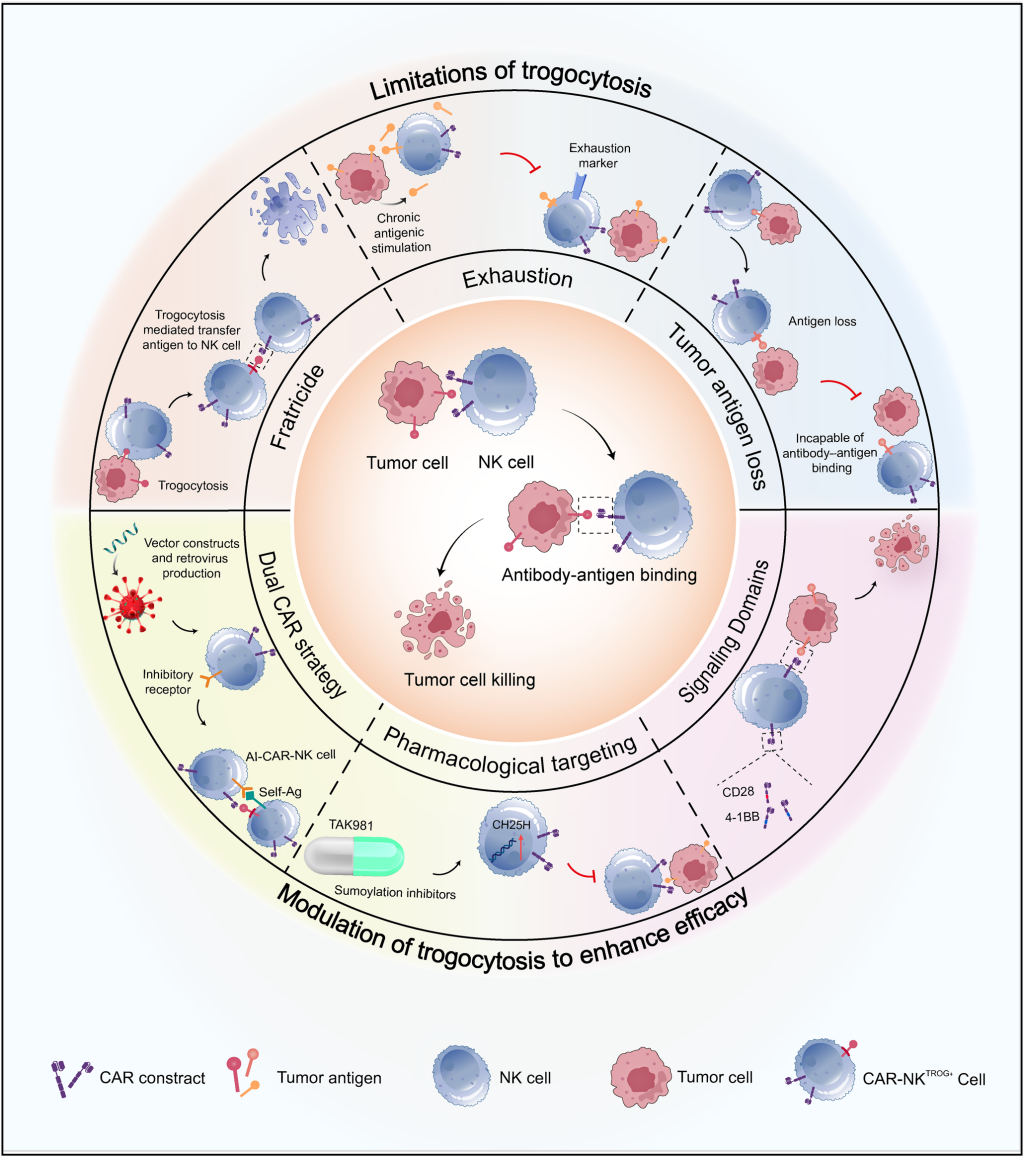
Graphical illustration of the challenges and potential enhancements associated with CAR‐NK cell therapy via trogocytosis. The upper segment illustrates the limitations of trogocytosis, highlighting issues such as fratricide, enhanced by trogocytosis‐mediated transfer of tumour cell antigens to NK cells, NK cell exhaustion marked by exhaustion markers, and antigen loss leading to the inability of antibody–antigen binding. The lower segment proposes potential strategies to augment the efficacy of the therapy, including a dual‐CAR strategy, pharmacological targeting utilising TAK981 to boost CH25H expression and modifications to signalling domains. CAR: chimeric antigen receptor; NK: natural killer; AI‐CAR: both activating chimeric antigen receptor and an inhibitory chimeric antigen receptor; CH25H: cholesterol 25‐hydroxylase; CD: cluster of differentiation.

Conversely, trogocytosis in NK cells may potentially diminish the efficiency of NK cell–based immunotherapeutic strategies. Halim and colleagues demonstrated through trogocytosis, NK cells could acquire PD‐1 from leukaemic cells, leading to an inhibitory NK cell antitumour response [56]. Chronic antigenic stimulation by tumour cells is widely known to lead to exhaustion in immune effector cells [57, 58, 59]. Li et al. illustrated that CAR activation facilitated the transfer of CAR cognate antigen from tumour cells to NK cells. This led to self‐recognition by antibodies to antigens on the surface of NK cells and repeated antigen‐mediated CAR activation, subsequently causing fratricide and exhaustion (Figure 3). This occurrence might be counteracted by employing a dual‐CAR system, which includes both an activating CAR targeting the cognate tumour antigen and an inhibitory CAR designed for NK cell self‐recognition. This system reduced fratricide mediated by trogocytic antigen transfer [60]. Trogocytosis can also lead to antigen loss on target cells, which has been demonstrated in studies on CAR‐NK engineering. As antigen density affects CAR functionality, the downregulation or internalisation of target antigens may lead to tumour evasion. Thus, strategies to counteract trogocytosis‐induced antigen loss could enhance tumour clearance. Modifying the CAR signalling domain may offer a potential solution to this problem, as trogocytosis has been shown to differentially impact CARs with CD28 or 4‐1BB signalling domains (Figure 3) [60, 61]. Potential strategies to modulate trogocytosis include pharmacological targeting, dual‐CAR systems and signalling domain modification [62]. Continued research in these areas will exert a substantial influence on progressing the modulation of trogocytosis, establishing it as a potent therapeutic approach in treating cancer.

#### 3.1.2 EP

In general, as a simple and cost‐effective way, EP is used to introduce nucleic acids. In several studies, non‐viral EP technologies have been used to transfect DNA or mRNA to enable transient protein and CAR expression (Figure 2C). DNA transfection is less efficient but allows for the easy introduction of complex constructs. With the self‐limiting nature of non‐integrated DNA, this transfection method offers an enhanced safety profile in comparison to viral methods. Ingegnere et al. introduced an innovative technique facilitating plasmid DNA efficient EP–based transfection, encompassing both CAR and CCR7 genes, into resting or cytokine‐expanded human NK‐cell populations, as well as the NK‐92 cell line [45]. This new technology allowed a significant improvement in the killing and migratory ability of NK cells, and the efficiency of EP with different sized plasmids.

In contrast to DNA transfection, mRNA transfection seems to be a more efficient, rapid and transient method for generating CAR‐NK cells. Technological advancements show that the substitution of high‐purity CAR mRNA for cDNA in a plasmid has significantly enhanced transfection efficiencies in NK cells, reaching as high as 90% with minimal adverse impacts on cell viability [63]. Boissel et al. revealed that EP using mRNA led to high expression of anti‐CD19‐CAR in the NK‐92 cell line [42]. Other researchers have reviewed the application of mRNA transfection for CAR EP in activated or expanded NK cells, showing that temporary expression of CD19‐CAR reached up to 60% [43]. Nonetheless, after transfection, transfusing these cells needed to be transfused back into patients within a 7‐day period due to the transient CAR expression. Carlsten and colleagues successfully transfected NK cells via Current Good Manufacturing Practice–compliant mRNA EP method, inducing swift and repeatable transgenic expression in CAR‐NK cells without negatively influencing cytotoxic function, viability and phenotype [64]. After transfection, CAR expression of NK cells could be sustained for 3–7 days at a high level [44]. In 2022, a new procedure introduced by Roex et al. was the construction of dual‐CAR NK‐92 cells specifically by creating NK‐92 cells expressing both BCMA‐CAR and CD19‐CAR. More significantly, the dual‐CAR NK‐92 cells demonstrated a killing capacity exceeding 60% against both single and dual antigen‐expressing cell lines, as well as primary tumour cells, which might be used as an ‘off‐the‐shelf’ tool to tackle the issue of relapse [48]. Moreover, CAR mRNA–engineered NK cells are expected to become a novel therapeutic approach in treating solid tumours. Some researchers constructed a CAR by fusing the extracellular domain of the NKG2D to DAP12 to improve the cytolytic activity of NK cells, highlighting a promising therapeutic potential for treating metastatic colorectal cancer [46].

#### 3.1.3 LNP

The modification of NK cells with mRNA has shown considerable promise in ACT in targeting ability and cytotoxicity (Figure 2E). The applications of ionizable LNP, delivering RNA into NK cells, are consistently expanded. An efficient LNP–based method for transferring CD19‐CAR mRNA into primary NK cells was demonstrated using charge‐altering releasable transporter transfection, imparting only a minimal effect on the cells' phenotype and function [65]. Golubovskaya et al. presented a novel CAR mRNA‐LNP technology designed for the efficient transfection of NK cells expanded from primary peripheral blood mononuclear cells, resulting in the generation of functional CAR‐NK cells. BCMA‐CAR mRNA and CD19‐CAR mRNA were encapsulated within LNPs, respectively, leading to CAR expression in NK cells at rates of 95% and 78% [51]. It was also shown that nanoparticles could efficiently introduce small interfering RNA (siRNA) into NK cells. Nakamura and colleagues developed an LNP, composed of CL1H6 (CL1H6‐LNP), for the efficient delivery of both siRNA and mRNA to NK‐92 cells, respectively [50, 66]. Douka and colleagues identified an ex vivo mRNA delivery technology to engineer NK cells [52]. The mRNA‐LNP formulations achieved high transfection efficiency and protein expression by refining lipid composition and microfluidic parameters in NK cells.

### 3.2 CRISPR‐Cas9

CRISPR‐Cas9 has been recognized as a powerful tool for gene editing [67]. This methodology entails introducing guide RNA and Cas9 protein into cells. CRISPR‐Cas9 has been applied to primary NK cells to disrupt the CD38 gene, with the goal of averting NK cell fratricide when used in conjunction with daratumumab (anti‐CD38) in view of the fact that CD38 is expressed on NK cells as well as on tumour cells from multiple myeloma (MM) and acute myeloid leukaemia (AML) [68]. In parallel with CRISPR‐Cas9, a homologous donor DNA template is transfected into the cell (Figure 2B) [69]. The DNA template, by superseding the targeted gene, makes it possible to integrate genes that amplify antitumour functions. This methodology was used successfully in primary T cells: The goal of employing CRISPR‐Cas9 was to target the TCR gene for the insertion of a CD19 CAR cassette. Expressing the CD19 CAR under the control of the endogenous TCRα promoter resulted in more persistent and significantly enhanced expression, leading to superior persistence of T cells [70]. Following this paradigm, it is conceivable to craft CAR‐NK cells with enhanced potency and longevity for improved therapeutic outcomes.

Furthermore, CRISPR‐Cas9 has been utilised on exhausted NK cells in TME [71]. A deeper understanding of various modes of senescence, exhaustion and anergy, such as diminished effector cytokine production, impaired cytotoxicity, presence of inhibitory cytokines and dysregulated receptor signalling found in TME, may be helpful to design strategies to bolster NK cell functionality [72]. Thus, utilising CRISPR‐Cas9 to engineer and reactivate NK cells presents a promising immunotherapeutic strategy. Stimulating the activating receptors of NK cells can also significantly enhance their functionality. For instance, using an engineered CRISPR‐Cas9 system to upregulate the expression of NKG2D ligand has been demonstrated to bolster the immune response against pathogenic cells effectively [73]. Moreover, utilising CRISPR‐Cas9 technology to disrupt inhibitory pathways and overcome the challenges posed by immune checkpoints presents a novel approach to enhance the effector functions of NK cells. Inhibiting the NK group 2A (NKG2A) could enhance the efficacy of NK cells in ACT [74]. Pomeroy and colleagues utilised CRISPR‐Cas9 to efficiently knock out the inhibitory signalling genes, such as ADAM17 and PD‐1 in NK cells. The results showed enhanced NK cell activity, cytokine production and cytotoxicity in these genetically modified NK cells [75]. Furthermore, Daher et al. suggested that removal of cytokine‐induced SH2–containing protein checkpoint, a negative regulator of IL‐15 signalling, could increase the fitness of CAR‐NK cells [76]. Concurrently, TIGIT and CD96 were identified as inhibitors of NK cell activity in other studies [77]. Huang et al. described an efficient CRISPR platform and performed knockout of inhibitory receptor genes, and achieved a high efficiency in the knockout process [78].

Nevertheless, it has been reported that genome editing using CRISPR‐Cas9 can trigger DNA damage responses mediated by p53 and lead to cell cycle arrest of human cells [79]. Additionally, when utilising the CRISPR‐Cas9 system for in vivo applications in clinical trials, it is crucial to consider the possibility of pre‐existing humoral and cell‐mediated adaptive immune responses to the CRISPR‐Cas9 system in humans [80]. Addressing these concerns is imperative to ensure the safe application of CAR‐NK cell therapy in patients.

### 3.3 High Integration Efficiency of Transfection With Transposons of CAR Engineering

Most non‐viral techniques enable the production of CAR‐NK cell engineering primarily through transient CAR expression. To minimise dependence on viral vectors while still obtaining durable gene modifications, transposon systems have surfaced as the most straightforward non‐viral gene delivery tools.

Transposons, which are repetitive DNA elements capable of moving and reintegrating into various loci within the genome, have been a focus of genetics since Barbara McClintock's pioneering discovery [81]. This ‘cut‐and‐paste’ mechanism has been leveraged for numerous genetic applications (Figure 2D) [82]. To improve safety in gene therapy applications, DNA transposons are often utilised via a two‐vector system. This system includes the transposon housing the gene of interest, which is surrounded by terminal inverted repeats and the transposase enzyme. Three principal transposon families are utilised for gene engineering [82]. Sleeping Beauty (SB) transposon, discovered by Zoltan Ivics from salmonid fish, which was reconstructed from an inactive transposon sequence belonging to the Tc1/mariner superfamily, stood as the pre‐eminent transposon gene‐transfer system extensively examined [83, 84, 85]. Through multiple iterations, this system evolved, culminating in the hyperactive transposase SB100X variant, which boasted a 100‐fold enhancement in activity in contrast to the first‐generation transposase. Meanwhile, with continuous system improvements, transposition efficiency has been greatly enhanced, rivalling viral vector–based methods for stable gene insertion [86, 87, 88]. PiggyBac (PB) transposon, sourced from the cabbage looper, has developed as a tool for gene transfer [89]. The PB system employs a transposition mechanism akin to that of the SB. Through optimisation of transposase/transposon vectors, its efficiency has seen considerable enhancement [90, 91]. Over time, it has been optimised and improved to a hyperactive version. The hAT family includes two notable members: the Tol2 transposon, originally identified from the Japanese medaka fish, and the subsequently discovered TcBuster (TB) transposon, discovered from the red flour beetle [92, 93]. TB has demonstrated significant activity in human cell lines like HEK‐293 with its transposition efficiency being on par with that of PB and Tol2 when the amount of transposon plasmid transfected was 500 ng, and it could also transpose at a relatively high frequency in HeLa cells [92, 94]. Gurney and colleagues embarked on producing transposon‐based donor‐derived, primary CAR‐NK cells combined with the TB system, producing integration of genes in clinically relevant levels and potential of expansion [95].

However, genotoxicity, with its capacity to lead to oncogenesis, stands as a critical risk in gene engineering. This was evidenced by past occurrences linked to retroviral transduction [96, 97]. Recent instances have included two patients who, after receiving CAR T‐cell therapy employing non‐viral PB transposon vectors, developed CAR T‐cell lymphoma [98]. Significantly, the integration pattern of the PB transposon system is similar to that of murine leukaemia virus retroviruses, demonstrating biased insertions in transcription start sites [99]. Conversely, the SB transposon system displayed a pattern of integration that was more random in comparison with viral vectors as well as non‐viral PB transposons. Thus, there is an expectation of a diminished genotoxicity risk when using the SB system for engineering CAR‐NK cells. The SB is under investigation in the first human clinical trials with SLAMF7 CAR‐T cells to treat MM and CAR engineering of cytokine‐induced killer cells for relapsed acute lymphoblastic leukaemia [100, 101].

## 4 Improvement of CAR‐NK Technology

### 4.1 CAR‐NK Manufacturing and Storage

The demonstrated safety, rapid response of NK cells and the potential for allogeneic application have collectively contributed to the growing endeavours in creating ‘off‐the‐shelf’ NK cell therapies for treating cancer. Nevertheless, the path to clinical application is hindered by several obstacles, including achieving clinical‐grade ex vivo expansion, enhancing in vivo persistence and storage of CAR‐NK cells. To surmount these hurdles and augment the efficacy of NK cell–based therapies, a suite of approaches is being employed, such as the engineering of induced pluripotent stem cell (iPSC)–differentiated CAR‐NK (iPSC‐NK) and the improvement of cryopreservation.

#### 4.1.1 iPSC‐NK Cell: A Novel Approach to Manufacture CAR‐NK Cells

Donor‐derived NK cells may have certain defects due to variability in their expansion and functional capacity. In a clinical context, each batch needs to undergo validation, resulting in a delay before patients receive a therapeutic infusion. Additionally, the limited potential for expansion presents a challenge in amplifying the efficacy of NK cells via genetic modification. The tactic of utilising a genetically engineered CAR‐NK‐92 cell line, with the capacity for expansion to large doses, has been undergoing clinical trials [102, 103]. Nonetheless, as a transformed cell line, NK‐92 faces limitations due to genetic instability, requiring irradiation to limit its lifetime activity for clinical use.

With a new source of NK cells, iPSC‐NK therapy offers a pathway to circumvent numerous challenges of NK cell therapy. iPSCs offer a homogeneously differentiated population of NK cells. They could be expanded to clinical scales and thereby address the limitations associated with the NK‐92 cell line. Some research groups have successfully shown that functional NK cells can be derived in vitro from iPSCs [104, 105, 106]. CAR–engineered iPSC‐NK cells have demonstrated their capability to effectively target human tumours [107]. Nevertheless, iPSC‐NK cells might cause tumorigenicity and also probably lead to genetic instability in prolonged in vitro culture conditions [108]. Further research is needed to address safety issues and enable large‐scale production.

#### 4.1.2 Improvement of Cryopreservation: Better Storage of NK Cells

Researchers have identified cryopreservation as a significant technical challenge in the study of NK cells. Authorities require large‐scale expansion of CAR‐NK cells under GMP conditions for clinical application and patient infusion. Given the cellular status and logistical preparations for the patient, administering the freshly expanded CAR‐NK cell product at the optimal time is virtually unfeasible [109]. Effective cryopreservation methods allow coordination of the therapy with patient care and completion of quality control and safety testing [110]. Traditional cryoprotective agents, like dimethyl sulfoxide (DMSO), are widely used, but impact cell biology, and exhibit noticeable toxicity [111]. The aftermath of using DMSO ranged from unwanted shifts in NK/T‐cell markers to diminished immune cell functionality, and even fatalities in certain clinical settings [112]. As the first CAR‐T therapies gained approval in the U.S., the demand for safer, DMSO‐free preservation methods has surged. The ideal cryopreservation method should be clinically safe, enable large‐scale production and facilitate direct infusion without pre‐infusion cell rest or culturing as identified by research. Addressing this challenge is critical to harness the full therapeutic potential of CAR‐NK cells. Several research teams have dedicated their efforts to refining the constituents of freezing medium and techniques to expand CAR‐NK cells, yielding promising results [109, 110, 113]. An innovative method using nanoparticle‐mediated intracellular protection has been introduced to prevent cryoinjury in NK cells and to retain their cytotoxic ability, potentially replacing DMSO as a cryoprotectant [114]. Additionally, IF‐M, a Me2SO‐free cryopreservation medium for CAR‐NK cells, significantly improved the viability, recovery and cytotoxic function of CAR‐NK cells post‐thaw, particularly over long‐term storage [115]. These encouraging results are likely to catalyse a paradigm shift in the storage and transport of therapeutic cells, broadening their accessibility and application in personalised medicine.

### 4.2 Emerging Technologies in Non‐Viral Delivery

Within the domain of cellular immunotherapies, NK cell–based therapy has not been investigated to the extent of T‐cell therapy, which indicates a relatively unexplored area for advancement. Several non‐viral intracellular delivery methods, which have shown encouraging outcomes in CAR‐T cells, remain to be applied to NK cells. Consequently, these non‐viral delivery mechanisms present viable research and development opportunities for enhancing NK cell therapy.

#### 4.2.1 Sonoporation

**FIGURE 4:**
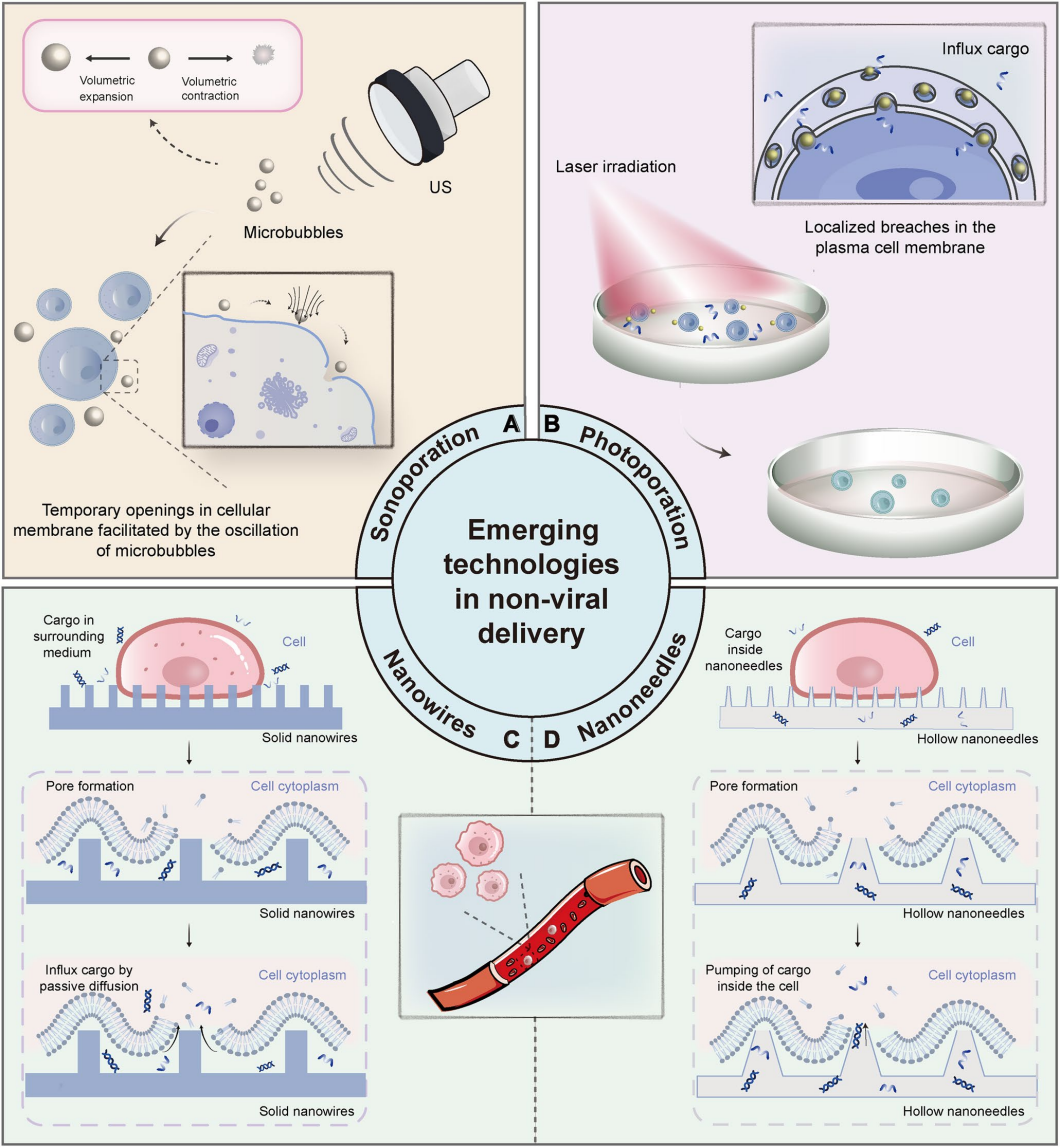
Mechanism of promising technologies in non‐viral delivery. (A). Sonoporation. Microbubbles expand or contract when subjected to US, which create temporary openings in cellular membrane, allowing an influx of external cargo molecules into the cytosol. (B). Graphical illustration of VNB photoporation. VNB–induced photoporation involves pretreating cells with photothermal agents, commonly gold nanoparticles, which attach to their exteriors, followed by pulsed laser exposure. The rapid expansion and subsequent collapse of these bubbles momentarily disrupt the cellular membrane, creating openings through which macromolecules, like mRNA, can enter the cytoplasm. (C). The figure illustrates nanowire‐mediated cargo delivery into the cell. Solid nanowires create pore formation in the cell membrane, allowing for the passive diffusion of extracellular cargo directly into the cytoplasm. (D). The figure illustrates hollow nanoneedles delivering for cargo into a cell by first creating membrane pores, and then pumping the cargo directly into the cytoplasm. US: ultrasound stimulation; VNB: vapour nanobubble.

Sonoporation, a physical permeabilisation method, holds significant potential and has proven effective for delivering diverse functional cargoes [116]. Sonoporation involves the creation of temporary openings in cellular membranes facilitated by the oscillation of microbubbles (Figure 4A). These microbubbles are composed of gaseous particles enveloped by a stabilising shell that oscillates when exposed to ultrasonic waves. Such oscillations can disturb the integrity of adjacent cellular membranes via multiple mechanisms, thereby enabling the entry of exogenous molecules into the cytosol [117, 118, 119, 120, 121]. For instance, sonoporation has been employed in the ex vivo introduction of pDNA into human haematopoietic stem and progenitor cells and siRNA into T cells [122, 123]. Another example is the research of De Timmerman and colleagues who designed mRNA‐lipoplexes attached to microbubbles for the transient transfection of dendritic cells [124]. In 2024, Wu et al. demonstrated that a clinically deployed ultrasound system, in conjunction with Phase 2–proven microbubbles, could ameliorate TME hypoxia and augment the effectiveness of immunotherapy in a murine model of pancreatic ductal adenocarcinoma [125]. The efficacy of sonoporation in the transfection of lymphocytes, monocytes and granulocytes was assessed by Duan et al. [126]. They discovered that lymphocytes, including NK cells, exhibited higher transfection efficiencies than granulocytes and experienced the lowest loss of viability. However, it was noted that lymphocytes displayed a reduction in size and granularity following sonoporation, prompting the researchers to fully understand the bio‐effects that could affect the functionality of engineered cells and comprehensively assess the viability of sonoporation within this field. It is also paramount to conduct a thorough investigation into the kinetics of pore formation and repair in sonoporation, the thermodynamics of acoustic forces on the cell membrane and any additional intracellular delivery pathways induced by sonoporation, such as endocytosis. These studies will offer vital insights to the sonoporation and intracellular delivery community, which might be the key technology for CAR‐NK engineering.

#### 4.2.2 Photoporation

Vapour nanobubble–mediated (VNB) photoporation offers a flexible and efficacious strategy for introducing various effector molecules into cells. This method hinges on the generation of VNBs triggered by the laser irradiation of photothermal nanomaterials, such as plasmonic gold nanoparticles. These nanomaterials are often designed to carry a cationic charge to facilitate electrostatic attachment to the cell membrane [127, 128]. Following attachment, the nanoparticles are exposed to laser pulses. The laser pulse absorption causes the nanoparticles to heat rapidly. Once the nanoparticles' temperature exceeds the critical threshold of water, VNBs form in the vicinity of the nanoparticles. Upon accumulating thermal energy, VNBs expand and then collapse when the energy is released. This process produces mechanical energy, manifesting as pressure waves, which in turn cause localised breaches in the plasma cell membrane, facilitating the entry of cargo molecules into the cytosol (Figure 4B). Recent advancements have shifted from the predominant use of non‐degradable inorganic nanoparticles as nanosensitisers towards more biodegradable and biocompatible alternatives, such as polydopamine nanoparticles, for the genetic modification of T cells [129]. Some researchers revealed that when unstimulated T cells underwent photoporation with polydopamine nanoparticles of 150 and 250 nm, they preserved their capacity for activation. In contrast, cells treated with 400 nm nanoparticles exhibited a diminished response. Their findings suggest that employing polydopamine nanoparticle‐photoporation for transfecting quiescent T cells is effective, but it requires the nanoparticles to be small enough to minimise cellular damage [130]. Alternatively, a strategy has been proposed wherein light‐sensitive iron oxide nanoparticles are embedded within electrospun nanofibres, serving as a substrate for cell culture [131]. After the transfection process, cells from the substrate were collected, leaving the nanoparticles firmly entrenched within the nanofibres. Crucially, studies on T cells have shown that photoporation employing these photothermal nanofibres induces fewer phenotypic alterations in contrast to EP, leading to an enhanced cytolytic capability of the cells. Therefore, photoporation emerges as a promising non‐viral transfection technology, holding significant potential for its application in NK cell engineering beyond its existing uses.

#### 4.2.3 Nanowires and Nanoneedles

An alternative method for inducing cell membrane permeabilisation involves the use of microfabricated nanowires and nanoneedles (NNs) [10, 132]. In this approach, cells are cultured or collected on substrates featuring sharp protrusions that facilitate piercing or enhancing the cell membrane's permeability. Characterised by their solid, needle‐like appearance, nanowires contrast with NNs, which are hollow and enable the direct infusion of cargo into cells from a reservoir situated below the nanowires (Figure 4C,D). Several published studies have applied these nanotechnologies to modify immune cells, including macrophages, T cells and dendritic cells [10, 133, 134]. Although not yet applied to NK cell engineering, Bhingardive and colleagues have developed nanowires for the chemical and mechanical stimulation of NK cells, with the goal of inducing their activation. By adjusting the diameter of the nanowires, they successfully generated two distinct phenotypes with different morphology and immune responses, which introduces a novel route for shaping NK cells in immunotherapy [135]. Chen et al. observed that polymeric NNs form close contact with cells, facilitating mRNA delivery to both adherent and nonadherent cell types (with transfection efficiencies of 49.4% ± 11% for GPE86 cells and 12% ± 7% for L1.2 cells). The effective nano‐injection achieved using polymeric NNs could potentially lower nanofabrication costs and provide opportunities for incorporating nanostructures into conventional polymeric cell cultureware for specific cellular manipulations [136].

### 4.3 Other Possible Improvements Based on Existing Methodologies

In the past decade, the adoptive transfer of engineered NK cells has attracted considerable attention. Novel strategies are still needed to enhance the clinical efficacy and safety of NK cell‐based immunotherapies. Several researchers have combined CAR‐NK therapy with other treatments to improve the efficacy of treating cancer. Focaccetti and colleagues provided a new approach in combination with DNAM‐1‐chimeric receptor–engineered NK cells and Nutlin‐3a for treating solid tumours carrying dysfunctional p53, such as human neuroblastoma [137]. Gurney described a non‐viral approach to generate primary CLL‐1 CAR‐NK cells through transposon engineering and CRISPR‐Cas9 genome editing, resulting in increased antigen‐specific activity against CLL‐1‐positive AML cell lines and primary AML populations [95]. Additionally, CAR engineering can also be combined with technologies used in other fields to create fresh cross‐fields, such as therapeutic antibodies and other cell therapies. To overcome difficulties associated with CAR delivery in solid tumours, various methods are being employed such as intraperitoneal administration, local administration and the application of focused ultrasound‐guided delivery. For example, pleural injection had a longer functional persistence compared to intravenous injection in an orthotopic model mimicking human pleural malignancies [138]. Furthermore, anti‐HER2 CAR‐NK‐92 cells have been recruited to the tumour site in the rodent brain using MRI–guided focused ultrasound and microbubbles [139]. To reduce the occurrence of poor therapeutic outcomes caused by the blood–brain barrier (BBB), CAR‐NK cells were transmitted into the brain via intravenous injection following interaction with focused ultrasound and microbubbles. This finding enabled the NK‐92 cells to pass through the BBB temporarily. Research comparing acoustofluidic and static ultrasound treatments indicates that acoustofluidic systems significantly enhance intracellular molecule delivery. This finding could facilitate continuous‐flow acoustofluidic cell transfection, which, when combined with sonoporation, may prove essential in cancer therapy [140]. Nano‐injection holds the potential for integration with EP in CAR‐NK cell engineering. Chen and colleagues have demonstrated its reliability in CAR T‐cell engineering with vertical electroactive nanotubes [141].

## 5 Prospect and Conclusion

The evolution of non‐viral technologies in CAR‐NK cell engineering is setting new standards for safety and efficiency in cancer immunotherapy. Looking ahead, the continued development and refinement of non‐viral gene delivery technologies hold great promise for advancing CAR‐NK cell therapies. By integrating innovative approaches such as 3D bioprinting, nanotechnologies and gene editing tools like zinc‐finger nucleases and transcription activator‐like effector nucleases, future research may address current limitations in cell viability, efficacy and large‐scale production [142, 143]. These advancements not only have the potential to improve therapeutic outcomes in challenging settings such as solid tumours but also pave the way for more accessible and personalised cancer treatments. Moreover, harnessing artificial intelligence (AI) to combine CAR‐NK cells holds tremendous potential in enhancing personalised medicine, particularly in the design and optimisation of CAR‐NK cell engineering. AI can facilitate the design of specific antibodies, optimise binding interactions and screen for the most effective therapeutic candidates [144]. By integrating AI into CAR‐NK engineering, personalised and highly targeted treatments can be developed, significantly advancing the precision and efficacy of cancer immunotherapy. Daniels et al. found that combining signalling motif libraries with machine learning could guide the design of non‐natural costimulatory domains with improved phenotypes [145], which might yield more diverse and nuanced phenotypic significance in CAR‐NK cells.

In the burgeoning field of cancer immunotherapy, non‐viral CAR‐NK cell therapy emerges as a promising avenue. The advantages and disadvantages of these transient and stable transfection technologies are in Table 3. Clinical trials of CAR‐NK cell therapy in treating tumours have also been carried out (Table 4). However, it still faces some obstacles. The primary pain points in the current landscape of non‐viral CAR‐NK cell engineering revolve around efficacy, storage and manufacturability. The transient nature of non‐viral gene delivery, although safer, results in brief CAR expression, necessitating multiple administrations to sustain therapeutic levels. Stable CAR‐NK cell engineering might be the future technology for achieving long‐lasting gene expression. Nonetheless, their applicability for CAR‐NK constructs remains to be fully elucidated. Innovations in cryopreservation techniques and the development of ‘off‐the‐shelf’ CAR‐NK cell products could significantly reduce treatment costs and increase accessibility for patients. In conclusion, while non‐viral CAR‐NK cell engineering presents a promising avenue for cancer treatment, realising its full potential requires overcoming significant scientific and technical challenges. Continued research and collaboration across the fields of immunology, genetic engineering and nanotechnology are imperative to harness the full therapeutic potential of CAR‐NK cells in the fight against cancer.

**TABLE 3:** Advantages and disadvantages of non‐viral technologies in CAR‐NK cells studies.

Technology	Advantages	Disadvantages
Trogocytosis	Enhances NK cell capabilities by acquiring functional molecules	Diminished efficiency due to acquisition of inhibitory molecules Antigen loss Chronic antigen stimulations cause exhaustion
EP	Simple and cost‐effective for nucleic acid introduction High transfection rates with mRNA	DNA transfection less efficient than mRNA Expression with mRNA transfection is transient, requiring quick application post‐transfection
LNP	High transfection rates Minimal impact on NK cell phenotype and function Efficient delivery of siRNA and mRNA	Technological complexity
CRISPR‐Cas9	Precise gene targeting	Potential for DNA damage responses Permanent modifications could pose risks if adverse reactions occur
Transposon systems	Attaining durable gene modifications without viral vectors	Risk of oncogenesis with certain systems (PB) Technical and resource‐intensive setup required

Abbreviations: CRISPR‐Cas9, clustered regularly interspaced short palindromic repeats–associated protein 9; EP, electroporation; LNP, lipid nanoparticle; NK, natural killer; PB, piggyBac; SB, Sleeping Beauty.

**TABLE 4:** CAR‐NK cell application in cancer treatments.

NCT Number	Study title	Study status	Conditions	Interventions
NCT05645601	CAR‐NK–targeted CD19 for R/R B‐cell malignancies	RECRUITING	Adult R/R B‐cell hematologic malignancies	BIOLOGICAL: CD19‐CAR‐NK
NCT05776355	NKG2D CAR‐NK and ovarian cancer	RECRUITING	Ovarian cancer	BIOLOGICAL: NKG2D CAR‐NK
NCT05213195	NKG2D CAR‐NK cell therapy in patients with refractory metastatic colorectal cancer	RECRUITING	Refractory metastatic colorectal cancer	DRUG: NKG2D CAR‐NK
NCT06045091	To evaluate the safety and efficacy of human BCMA–targeted CAR‐NK cells injection for subjects with R/R MM or PCL	RECRUITING	MM|PCL	DRUG: human BCMA–targeted CAR‐NK cells injection
NCT05574608	Allogenic CD123‐CAR‐NK cells in the treatment of R/R AML	RECRUITING	AML refractory|AML recurrent	BIOLOGICAL: CD123‐CAR‐NK cells
NCT05410041	Anti‐CD19 CAR–engineered NK cells in the treatment of R/R B‐cell malignancies	RECRUITING	Acute lymphocytic leukaemia|chronic lymphocytic leukaemia|NHL	BIOLOGICAL: CAR‐NK‐CD19 cells
NCT05739227	Safety and efficacy of allogenic CD19‐CAR‐NK cells in treating R/R B‐cell hematologic malignancies	RECRUITING	Acute lymphoblastic leukaemia|B‐cell lymphoma|chronic lymphocytic leukaemia	OTHER: allogenic CD19‐CAR‐NK cells
NCT05507593	Study of DLL3‐CAR‐NK cells in the treatment of extensive stage small cell lung cancer	RECRUITING	SCLC, extensive stage	BIOLOGICAL: DLL3‐CAR‐NK cells
NCT05410717	CLDN6/GPC3/mesothelin/AXL‐CAR‐NK cell therapy for advanced solid tumours	RECRUITING	Stage IV ovarian cancer|testis cancer, refractory|endometrial cancer recurrent	BIOLOGICAL: CLDN6, GPC3, mesothelin, or AXL–targeting CAR‐NK cells
NCT05673447	The study of anti‐CD19 CAR‐NK cells in the treatment of R/R diffuse large B‐cell lymphoma	RECRUITING	Diffuse large B‐cell lymphoma	BIOLOGICAL: anti‐CD19 CAR‐NK cells
NCT04887012	Clinical study of HLA haploidentical CAR‐NK cells targeting CD19 in the treatment of R/R B‐cell NHL	RECRUITING	B‐cell NHL	BIOLOGICAL: anti‐CD19 CAR‐NK
NCT05652530	Clinical study of the safety and efficacy of BCMA CAR‐NK	RECRUITING	Immunotherapy|MM	DRUG: CAR NK‐cell injection targeting BCMA
NCT05472558	Clinical study of cord blood–derived CAR‐NK cells targeting CD19 in the treatment of R/R B‐cell NHL	RECRUITING	B‐cell NHL	BIOLOGICAL: anti‐CD19 CAR‐NK
NCT06006403	Safety and efficacy of CD123–targeted CAR‐NK for R/R AML or BPDCN	RECRUITING	AML|BPDCN|relapse leukaemia|refractory leukaemia	BIOLOGICAL: CD123–targeted CAR‐NK cells
NCT05570188	Anti‐CD19 universal CAR‐NK cells therapy combined with HSCT for B‐cell hematologic malignancies	WITHDRAWN	B‐cell lymphoma|B‐cell leukaemia	BIOLOGICAL: anti‐CD19 UCAR‐NK cells
NCT05845502	Single‐arm, open‐label clinical study of SZ003 in the treatment of advanced hepatocellular carcinoma	NOT_YET_RECRUITING	Advanced hepatocellular carcinoma	BIOLOGICAL: SZ003 CAR‐NK
NCT06066424	Phase 1 dose escalation and expansion study of TROP2 CAR–engineered IL‐15–transduced cord blood–derived NK cells in patients with advanced solid tumours (TROPIKANA)	RECRUITING	Solid tumours	DRUG: rimiducid|DRUG: TROP2‐CAR‐NK cells|DRUG: fludarabine phosphate|DRUG: cyclophosphamide
NCT05922930	Study of TROP2 CAR–engineered IL‐15–transduced cord blood–derived NK cells delivered intraperitoneally for the management of platinum resistant ovarian cancer, mesonephric‐like adenocarcinoma, and pancreatic cancer	RECRUITING	Pancreatic cancer|ovarian cancer|adenocarcinoma	DRUG: TROP2‐CAR‐NK|DRUG: cyclophosphamide|DRUG: fludarabine
NCT05110742	Phase I/II study of CD5 CAR–engineered IL‐15–transduced cord blood–derived NK cells in conjunction with lymphodepleting chemotherapy for the management of R/R haematological malignances	NOT_YET_RECRUITING	Haematological malignancy	DRUG: fludarabine phosphate|DRUG: cyclophosphamide|DRUG: CAR.5/IL‐15–transduced cord blood‐NK cells
NCT06242249	Anti‐BCMA CAR‐NK therapy in R/R MM	NOT_YET_RECRUITING	Determining safety and MTD of anti‐BCMA CAR‐NK therapy in R/R MM	BIOLOGICAL: anti‐BCMA CAR‐NK
NCT04847466	Immunotherapy combination: Irradiated PD‐L1 CAR‐NK cells plus pembrolizumab plus N‐803 for subjects with recurrent/metastatic gastric or head and neck cancer	RECRUITING	GEJ cancers|advanced HNSCC	DRUG: N‐803|DRUG: pembrolizumab|BIOLOGICAL: PD‐L1 t‐haNK
NCT05008575	Anti‐CD33 CAR‐NK cells in the treatment of R/R AML	UNKNOWN	AML	BIOLOGICAL: anti‐CD33 CAR‐NK cells|DRUG: fludarabine|DRUG: cytoxan
NCT04623944	NKX101, intravenous allogeneic CAR‐NK cells, in adults with AML or MDS	RECRUITING	R/R AML|AML, adult|MDS|refractory MDS	BIOLOGICAL: NKX101–CAR‐NK cell therapy
NCT06201247	‘Off‐the‐shelf’ CD123 CAR‐NK for R/R AML	RECRUITING	AML, in relapse| AML refractory	DRUG: JD123 injection
NCT05092451	Phase I/II study of CAR.70–engineered IL‐15–transduced cord blood–derived NK cells in conjunction with lymphodepleting chemotherapy for the management of R/R haematological malignances	RECRUITING	B‐cell lymphoma|MDS|AML	DRUG: cyclophosphamide|DRUG: CAR.70/IL‐15–transduced cord blood–NK cells|DRUG: fludarabine phosphate
NCT05941156	Clinical study of anti‐CD56‐CAR‐T in the treatment of R/R NK/T‐cell lymphoma /NK‐cell leukaemia	RECRUITING	Extranodal NK T‐cell lymphoma|NK‐cell leukaemia	BIOLOGICAL: anti‐CD56 CAR‐T
NCT05667155	Clinical study of cord blood–derived CAR‐NK cells targeting CD19/CD70 in R/R B‐cell NHL	RECRUITING	B‐cell NHL	BIOLOGICAL: cord blood dual‐CAR‐NK19/70
NCT05654038	A study of universal CD19–targeted UCAR‐NK cells combined with HSCT for B‐cell hematologic malignancies	RECRUITING	B‐cell lymphoblastic leukaemia/lymphoma	BIOLOGICAL: anti‐CD19 UCAR‐NK cells
NCT04796688	Universal CAR–modified AT19 cells for CD19+ R/R haematological malignancies	RECRUITING	Acute lymphoblastic leukaemia|chronic lymphoblastic leukaemia|B‐cell lymphoma	DRUG: fludarabine + cyclophosphamide + CAR‐NK‐CD19 cells
NCT05182073	FT576 in subjects with MM	RECRUITING	MM|myeloma	DRUG: FT576 (allogenic CAR‐NK cells with BCMA expression)|DRUG: cyclophosphamide|DRUG: fludarabine|DRUG: daratumumab|DRUG: bendamustine
NCT05703854	Study of CAR.70–engineered IL‐15–transduced cord blood–derived NK cells in conjunction with lymphodepleting chemotherapy for the management of advanced renal cell carcinoma, mesothelioma and osteosarcoma	RECRUITING	Advanced renal cell carcinoma|advanced mesothelioma|advanced osteosarcoma	DRUG: CAR.70/IL‐15–transduced cord blood–derived NK cells|DRUG: fludarabine phosphate|DRUG: cyclophosphamide
NCT05487651	Allogeneic NK T cells expressing CD19‐specific CAR in B‐cell malignancies	RECRUITING	NHL, relapsed, adult|B‐cell lymphoma|B‐cell leukaemia|DLBCL—diffuse large B cell lymphoma|ALL, adult B cell|ALL, childhood|CLL/SLL	GENETIC: KUR‐502
NCT05686720	Single‐arm, open‐label clinical study of SZ011 in the treatment of advanced triple‐negative breast cancer	NOT_YET_RECRUITING	Advanced triple‐negative breast cancer	DRUG: SZ011 CAR‐NK
NCT05856643	Single‐arm, open‐label clinical study of SZ011 in the treatment of ovarian epithelial carcinoma	NOT_YET_RECRUITING	Ovarian epithelial carcinoma	DRUG: SZ011 CAR‐NK
NCT05842707	Study of cord blood–derived CAR‐NK cells targeting CD19/CD70 in R/R B‐cell NHL	RECRUITING	R/R B‐cell NHL	DRUG: dual‐CAR‐NK19/70 cell
NCT04538599	RD13‐01 for patients with R/R CD7+ T/NK cell hematologic malignancies	COMPLETED	Hematologic malignancies	DRUG: RD13‐01 cell infusion
NCT04264078	Anti‐CD7 U‐CAR T‐cell therapy for T/NK cell hematologic malignancies	UNKNOWN	T‐cell leukaemia|T‐cell lymphoma	BIOLOGICAL: CD7 UCAR‐T cells|DRUG: fludarabine|DRUG: cytoxan|DRUG: melphalan
NCT05995028	Universal 4SCAR7U targeting CD7–positive malignancies	RECRUITING	T‐cell acute lymphoblastic leukaemia|T‐cell acute lymphoblastic lymphoma|AML|NK cell lymphoma	BIOLOGICAL: universal CD7‐specific CAR gene–engineered T cells
NCT05336409	A study of CNTY‐101 in participants with CD19–positive B‐cell malignancies	RECRUITING	R/R CD19–positive B‐cell malignancies|indolent NHL |aggressive NHL	BIOLOGICAL: CNTY‐101|BIOLOGICAL: IL‐2|DRUG: lymphodepleting chemotherapy

Abbreviations: AML, acute myeloid leukaemia; BCMA, B‐cell maturation antigen; CAR, chimeric antigen receptor; CLDN6, Claudin‐6; GEJ, gastroesophageal junction; GPC3, Glypican 3; HSCT, haematopoietic stem cell transplantation; MDS, myelodysplastic syndromes; MM, multiple myeloma; MTD, maximum‐tolerated dose; NHL, non‐Hodgkin lymphoma; NK, natural killer; NKG2D, natural killer group 2D; PCL, plasma cell leukaemia; R/R, relapsed or refractory; SCLC, small cell lung cancer.

## Author Contributions

Z.Q.L., Z.K.Z. and X.W.H. provided guidance throughout the preparation of this manuscript. Y.F.C. and Z.K.Z. wrote the manuscript. Z.K.Z., Y.H.B., Y.Y.Z., L.L.Z., S.S.Z., X.Z., C.H.Z. and Y.K.C. reviewed and made significant revisions to the manuscript. T.C., Y.Q.R., Q.C., P.L., H.X., S.Y.W., A.N.Z. and S.T.L. revised the manuscript. Z.K.Z. and Y.F.C. collected and prepared the related papers. All authors read and approved the final manuscript.

## Conflicts of Interest

The authors declare no conflicts of interest.

## Data Availability

Data sharing not applicable to this article as no datasets were generated or analysed during the current study.
